# WHAT PANDEMIC RECOVERY WILL LOOK LIKE FOR OLDER, MULTICULTURAL WORKERS

**DOI:** 10.1093/geroni/igac059.1015

**Published:** 2022-12-20

**Authors:** Cassandra Burton, Katherine Bridges, Aisha Cozad

## Abstract

It is no surprise to many that the impact of Covid-19 on older adults, particularly LGBTQ and people of color has been detrimental, not just physically, but mentally, socially, and economically. Many African American/Black adults have suffered disproportionately during the pandemic. In terms of Social Security more than nine in 10 (93%) older Black Americans report that having adequate Social Security benefits is important, but for the many who were forced to retire early due to the pandemic, they will be at a disadvantage. LGBTQ older adults experience persistent discrimination due to sexual orientation or gender identity. When the LGBTQ individual was African American/Black, experiences of discrimination were higher and often occurred in the workplace. These forms of discrimination are directly impacts ones earning potential both during active work years and in retirement savings. AARP has a long history of being an advocate for marginalized and vulnerable adults. AARP staff will discuss policy needs and what the post pandemic workplaces needs to ensure that older LGBTQ people can thrive in the workplace with dignity and respect. The 2021 AARP’s Vital Voices research will be used to showcase the economic impact the pandemic has had on older adults, African American communities, Hispanic/Latino communities, Asian Pacific Islander communities and LGBTQ communities. AARP staff will discuss strategies and tactics needed to ensure that opportunities for economic recovery for older adults. The survey gathers information to gauge opinions on a range of topics as well as breaking and current issues.

